# Adult zebrafish infected by clinically isolated *Klebsiella pneumoniae* with different virulence showed increased intestinal inflammation and disturbed intestinal microbial biodiversity

**DOI:** 10.1186/s12879-023-08766-z

**Published:** 2023-12-21

**Authors:** Xu Wang, Ting Li, Lu Zhou, Fan Tu, Xiaohong Rui, Ze Xu, Jun Liu, Futao Cao

**Affiliations:** 1 Department of Laboratory Medicine, Affiliated Wuxi Fifth Hospital of Jiangnan University, Wuxi, Jiangsu 214005 China; 2 Department of Laboratory Medicine, Wuxi No.8 People’s Hospital, Wuxi, Jiangsu 214005 China; 3 Department of Laboratory Medicine, Jiangsu Provincial Center for Disease Control and Prevention, Nanjing, Jiangsu 210009 China; 4 Department of Laboratory Medicine, Wuxi Center for Disease Control and Prevention, Wuxi, Jiangsu 214005 China; 5 Emergency Department, Jiangnan University Medical Center, Wuxi, Jiangsu 214000 China

**Keywords:** Klebsiella pneumonia, Zebrafish, Intestine, Inflammation, Microbial diversity

## Abstract

**Background:**

Klebsiella pneumoniae is a pathogen that often infects patients in clinical practice. Due to its high virulent and drug resistance, infected patients are difficult to treat. In clinical practice, Klebsiella pneumoniae can infect patients' intestines, intestines, blood, etc., causing pathological changes. However, there is relatively little information on the impact of Klebsiella pneumoniae on intestinal inflammation and microbial populations. Zebrafish is an excellent biomedical model that has been successfully applied to the virulence assessment of Klebsiella pneumoniae.

**Methods:**

In this study, three clinically isolated representative strains of Klebsiella pneumoniae (high virulence non-resistant, high virulence resistant, and low virulence resistant) were used to infect zebrafish, and their effects on intestinal colonization, inflammation, pathology, and microbial diversity were tested.

**Results:**

Enzyme-linked immunoassay (ELISA) showed that Klebsiella pneumoniae significantly increased levels of the cytokines interleukin-1α (Il-1α), interleukin-1β (Il-1β), and tumor necrosis factor-α (Tnf-α), which increased inflammatory symptoms. Hematoxylin eosin staining(H&S) showed that Klebsiella pneumoniae treatment caused intestinal lesions in zebrafish, in which KP1053 exposure significantly decreased the number of goblet cells, KP1195 caused epithelial dissolution and exfoliation. In addition, Klebsiella pneumoniae disturbed the composition of intestinal microbiota, and the Shannon index increased, which increased the number of harmful bacteria.

**Conclusions:**

Klebsiella pneumoniae infection can lead to intestinal colonization, inflammation, pathological changes, and changes in microbial biodiversity. This study provides a reference for the intestinal pathology of clinical Klebsiella pneumoniae infection.

**Supplementary Information:**

The online version contains supplementary material available at 10.1186/s12879-023-08766-z.

## Background

*Klebsiella pneumoniae* is a gram-negative bacterium that widely exists in nature; however, it may lead to clinical infections and may cause serious effects [1]. Recent studies have found that in hospitals and communities, *K. pneumoniae* is highly infectious. In addition, patients in the intensive care unit (ICU) are more susceptible to infection, which may even lead to patient death [2]. Early reports on *K. pneumoniae* showed that the microbe is not very virulent. However, since the first case of highly virulent *K. pneumonia* was reported in Taiwan, highly virulent strains have become very common [3]. In addition, because of the abuse of antibiotics in the past ten years and the mutagenic characteristics of *K. pneumoniae* strains, highly virulent and drug resistant have evolved, which has become a very difficult clinical problem [4].

The virulence of *K. pneumoniae* is mainly caused by lipopolysaccharides, capsules, fimbriae, and the iron transport system [5]. Mice [6], rats [7], rabbits [8], and *Galleria mellonella* [9] can all be used as model organisms to assess the virulence of *K. pneumoniae*. Recently, zebrafish has become a relatively good model organism and has been successfully applied to the field of biomedicine and for clinical microbial virulence assessment. Zebrafish embryos, larvae fish, and adult fish can be used as pathogenic microorganism infection research models. Numerous pathogenic microorganisms such as *Streptococcus iniae* [10], *Streptococcus pyogenes* [11], *Salmonella typhimurium* [12], and *Snakehead rhabdovirus* [13] have been used to develop zebrafish models, and considerable research progress has been made. In 2018, Andrés et al. first established a zebrafish *K. pneumoniae* infection model [14]. In 2019, Zhang et al. used zebrafish to evaluate the virulence of two clinically isolated *K. pneumoniae* strains [15]. Zebrafish has since become a relatively excellent host organism to study *K. pneumonia* virulence and pathogenic mechanism. In addition, zebrafish can be used for high-throughput drug screening. As a result, Lalitha et al. in 2017 used zebrafish to screen active molecular drugs for the treatment of *K. pneumoniae* infection [16].

The intestine is the main digestive organ and one of the main organs for colonization of *K. pneumoniae* [17]. Its strains can be isolated from clinical intestinal mucosa. After infection, it can cause intestinal diseases such as intestine inflammation [17]. The homeostasis and diversity of microorganisms in the intestine are necessary for the intestine to maintain its normal function [18]. Recent research has shown that many diseases such as diabetes, inflammation, cardiovascular diseases, and even cancer, are accompanied by changes in intestinal microbial populations [19]. However, few reports on the homeostasis and diversity changes of the intestinal microbial population after *K. pneumoniae* infection have been published.

In order to understand whether the colonization of *K. pneumoniae* in the intestine is related to its virulence, and whether it has adverse effects on the intestine, three clinically isolated *K. pneumoniae* strains, which are high virulence non-drug-resistant (KP1053), high virulence drug resistant (KP1196), and low virulence drug resistant (KP1195) strains, were used. In this study, *K. pneumoniae* strains were injected into adult zebrafish to detect changes in intestinal inflammation and microbial colonial diversity. This study provides a reference for clinical analysis of *K. pneumoniae* intestinal pathology.

## Methods

### *Klebsiella pneumoniae* clinical isolates and patient information

The strain KP1053 was isolated from the ocular pus of an 80-year-old Chinese male patient. The patient had a history of type II diabetes, hypertension, coronary heart disease, and cataracts. The blood culture of the patient, pus aspirated from the liver abscess, and eyeball contents all produced *K. pneumoniae*, and the strains were sensitive to antibiotics. The same strain of bacteria (ST1764) were detected in all three parts of the body using multi-locus sequence typing (MLST). Strains KP1196 and KP1195 were isolated from the drainage fluid of a 36-year-old patient whose underlying disease was type II diabetes. *K. pneumoniae* was isolated from the blood and abdominal drainage fluid of the patient. After MLST testing, all isolates were found to be K64-ST11. Two carbapenem-resistant *K. pneumoniae* appeared in the drainage fluid in the abdominal cavity; however, their morphologies differed. Thus, the drug sensitivity test showed that they were all carbapenem-resistant. The virulence gene detection shows that KP1196 is a high-virulence strain, and KP1195 is a low-virulence strain (The data of about *Klebsiella pneumoniae* not shown).

### Fish maintenance

Adult zebrafish used in the experiment were cultured in a circulatory system with a constant temperature of 28 °C, with 14 h of light and 10 h of darkness. The zebrafish were fed three times a day with brine shrimp in the morning and evening, and commercial feed at noon. Five-month-old adult female zebrafish were infected with the isolated *K. pneumoniae*. The detailed infection method was based on a previous study [16]. Infected zebrafish were cultured in a 10 L glass tank, and fed brine shrimp in the morning and evening; the uneaten food and dead zebrafish were removed in time. The zebrafish that died every day were recorded, and their mortality was counted.

### Sample collection

Untreated zebrafish served as the control group and injected *K. pneumoniae* zebrafish served as the experimental group. In each set of experiments, a total of thirty fish were raised evenly in three separate tanks. Forty-eight hours after infection, the zebrafish were anesthetized with MS-222 and sacrificed by removing the spine before dissecting the intestinal tissue. Using a sterile scalpel, the entire intestine was removed under aseptic conditions. Nine fish in each group (three fish from each tank) were randomly selected to take the middle intestinal segment and obtain HE slices for intestinal histopathological analysis and colony diversity experiment. The tissue material (gut) of the fish used for bacterial recycling is the same (gram tissue). Six whole intestines (two fish from each tank) were randomly collected from each group for ELISA analysis, and nine whole intestines (three fish from each tank) were randomly collected from each group for DNA extraction.

### Histological analysis

For intestinal histopathological analysis, the intestinal tissue was washed with phosphate buffered saline (PBS), fixed in 10% neutral buffered formalin (10 mL/fish) at 4 °C for 24 h, dehydrated with ethanol, buried in paraffin, cut to a thickness of 4 µm, and then, stained with HE. Then, the intestinal section was observed under an optical microscope.

### Inflammatory factor analysis by enzyme-linked immunosorbent assay (ELISA)

The intestine was homogenized in 0.5 mL of 0.9% sodium chloride using a tissue homogenizer. The homogenate was centrifuged at 3000 rpm at 4 °C for 10 min to obtain the supernatant for cytokine analysis. Following this, ELISA was performed to measure the interleukin-1α (Il-1α), interleukin-1β (Il-1β), and tumor necrosis factor-α (Tnf-α) levels in fish according to manufacturer’s instructions. All samples and standards were repeated three times.

### Genomic DNA extraction and Illumina high-throughput sequencing of barcoded 16S rRNA genes

The bacterial genomic DNA was extracted using a MoBio PowerSoil DNA 153 Isolation Kit (Mo Bio Laboratories Inc., California, USA) following the manufacturer’s protocol. The quantity and concentration of genomic DNA were measured and evaluated, as previously reported [20].

Genomic DNA was used for amplification of the 16S rRNA targeting the V3-V4 region. Amplicon sequencing was performed on an Illumina HiSeq 2500 platform (Zhejiang Tianke High Technology Development Co. Ltd, China). Raw data obtained by sequencing contained the linker and barcode sequences. These reads had to be removed before information analysis. Next, reads with overlaps were stitched using FLASH software, and the mosaic data were filtered using QIIME (Version 1.9.1) to filter out N-containing or low-mass sequences and finally chimera filtration to obtain valid data (effective tags) for subsequent analysis. The effective tags for all samples were clustered using UPARSE software (http://www.drive5.com/uparse/); the sequences were clustered into operational taxonomic units (OTUs) with 97% identity, and then, the OTUs representing the species sequences were annotated. According to species annotations, the number of sequences annotated to each classification level (kingdom, phylum, class, order, family, and genus) were counted for each sample. Alpha diversity indexes were calculated in QIIME from rarefied samples using the Shannon index for diversity and the Chao1 index for richness. Beta diversity was calculated using non-metric multi-dimensional scaling (NMDS). The unweighted paired group method with arithmetic mean (UPGMA) trees was built from the beta diversity distance matrices. For high-throughput sequencing, three bioreplicates were used per group, with three measurements per bioreplicate.

### Data analysis and statistics

The statistical significance levels were evaluated using one-way ANOVA followed by Dunnett’s post-hoc test using the StatView 6.0 program. Values were considered significantly different when the probability (p) was < 0.05. All data are presented as mean ± standard error of the mean (SEM).

## Results

### Survival status of zebrafish after infection of *K. pneumoniae* with different virulence

**Fig. 1:**
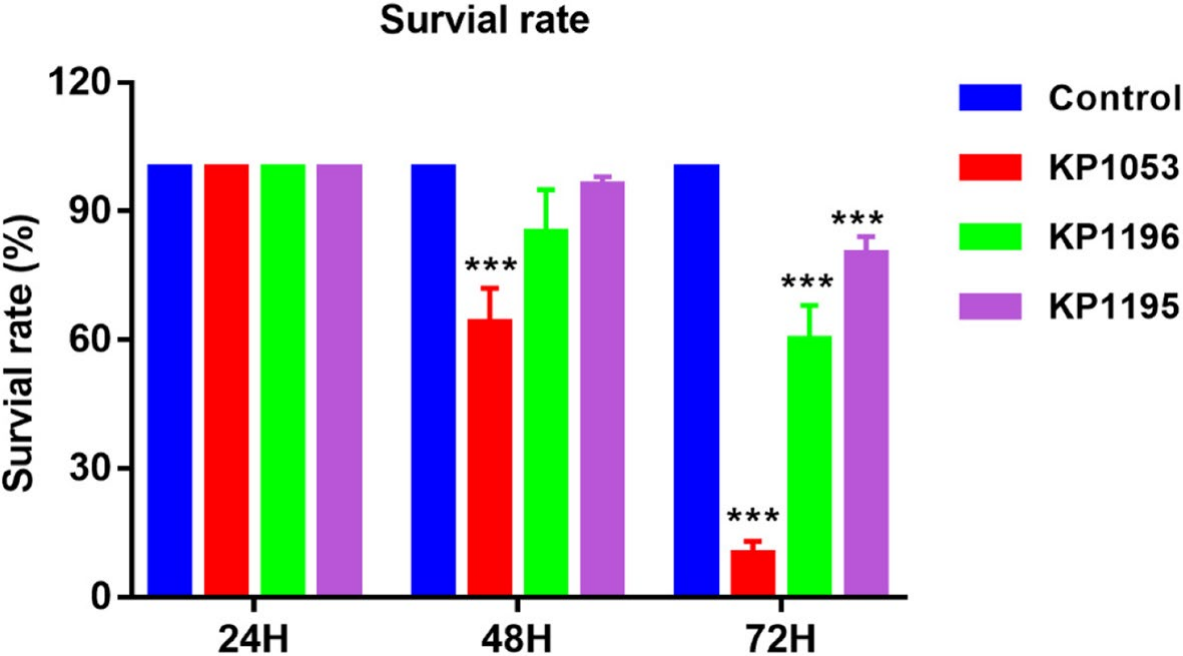
Zebrafish survival rate 24, 48 and 72 h after *Klebsiella pneumoniae* infection with different virulence. Error bars are presented as the SEM. Values are presented as the mean ± SEM of three replicates ( ****P < 0.0001)

Using different clinically isolated *K. pneumoniae* strains to infect normal zebrafish, as Fig. 1 showed, no significant difference in the survival status was observed 24 h after infection. After 48 h of infection, the survival rate of zebrafish infected by the KP1053 strain decreased significantly, whereas that of zebrafish infected by KP1196 and KP1195 did not show a significant decrease in survival rate. After 72 h of infection, the survival rate of zebrafish in the three infection groups decreased significantly. In the KP1053 infection group, almost all zebrafish died. These data show that these three groups of bacteria can significantly affect the survival rate of zebrafish.

We examined the virulence genes of three *K. pneumoniae*, and the results showed that _c_rmpA2 was missing in KP1053 and KP1196, and more seriously, five virulence genes were missing in KP1195, namely _p_rmpA, _p_rmpA2, iroB, icuA and fimH (Supplementary Table 1). In addition, three types of *K. pneumoniae* resistance showed that KP1053 was sensitive, and KP1195 and KP1196 were resistant (Data not shown).

### Colonization of the intestinal tract after infection with the three *K. pneumoniae* strains

**Fig. 2:**
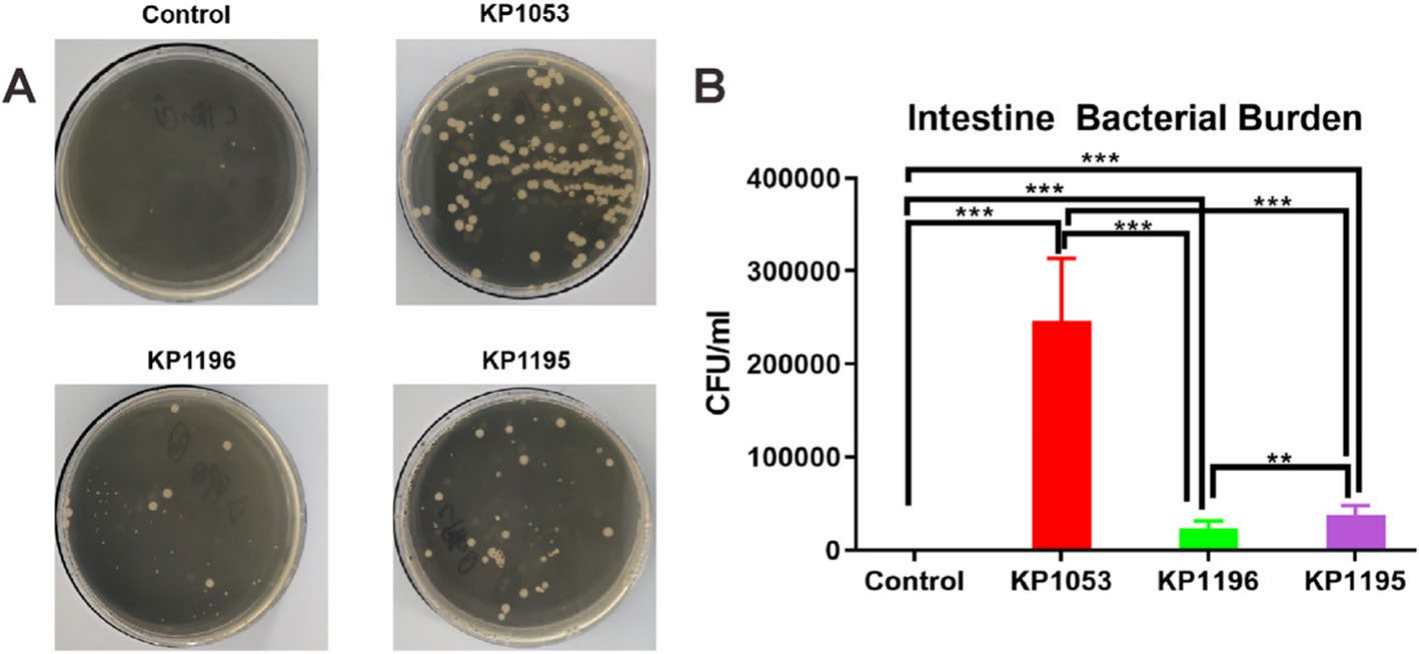
Analysis of intestinal colonization of zebrafish 48 h after *Klebsiella pneumoniae* infection with different virulence. **A** Representative plate in each groups. **B** CFU statistics in the intestines of zebrafish infected by *Klebsiella pneumoniae* with different virulence. Error bars are presented as the SEM. Values are presented as the mean ± SEM of three replicates (**P < 0.01, ***P < 0.001,)

To study the colonization of the zebrafish intestines after infection with the three strains of *K. pneumoniae*, the bacterial burden of the zebrafish intestines 48 h after infection was analyzed (Fig. 2A). The results showed that no *K. pneumoniae* strains were observed on the plate of the control group. *Klebsiella pneumoniae* colonies were formed in the extracts from the zebrafish intestines infected by the three strains of *K. pneumoniae*. The capacity of the burden of the three bacteria in intestines colonization is KP1053 > KP1195 > KP1196 (Fig. 2B). These data indicate that these three groups of *K. pneumoniae* can colonize zebrafish intestines.

### Changes in the intestinal structure after infection with the three strains of *K. pneumoniae*

**Fig. 3:**
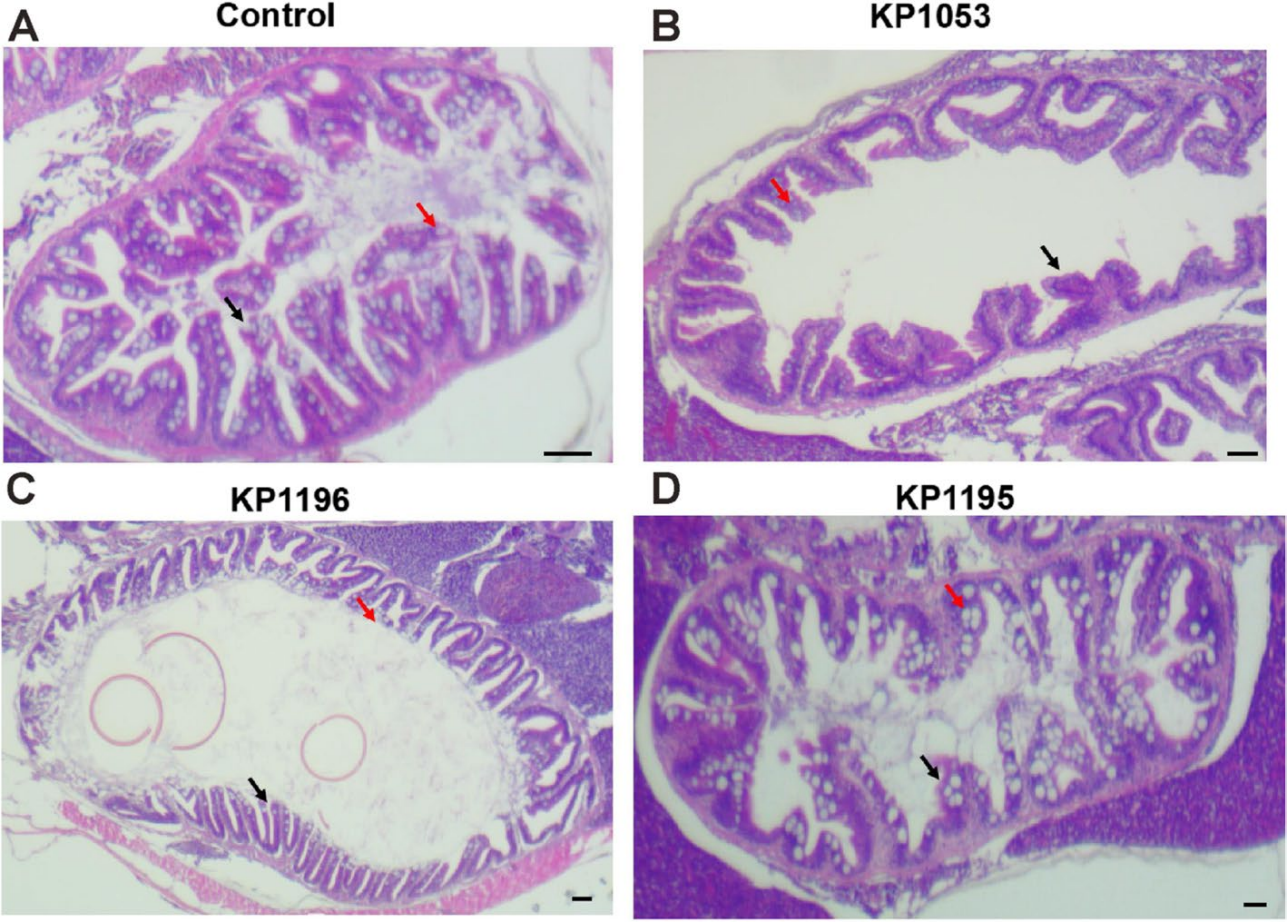
Changes in the structure of the gut 48 h after *Klebsiella pneumoniae* infected zebrafish with different virulence. The intestinal tract after infection was subjected to hematoxylin and eosin staining. **A** Control, (**B**) KP1052. **C** KP1196, and (**D**) KP1195. The red arrow indicates was goblet cells and the white arrows was epithelial cells. scale bars: 10μm

Hematoxylin and eosin staining was used to detect the changes in the intestinal tissue structure of the three groups of intestines infected by *K. pneumoniae* after 48 h of infection. Compared with the control group, the intestinal epithelium of the KP1053 bacterial infection group was significantly damaged, the intestinal ridge was significantly reduced, and the number of goblet cells was significantly reduced (Fig. 3A, B, Supplementary Fig. 1). In the KP1196 and KP1195 infection groups, tissue damage was significantly smaller (Fig. 3C, D). However, in the KP1195 infection group, the dissolved and shed epithelial cells were observed.

### Changes in inflammatory factor contents after three strains of Klebsiella pneumoniae

**Fig. 4:**
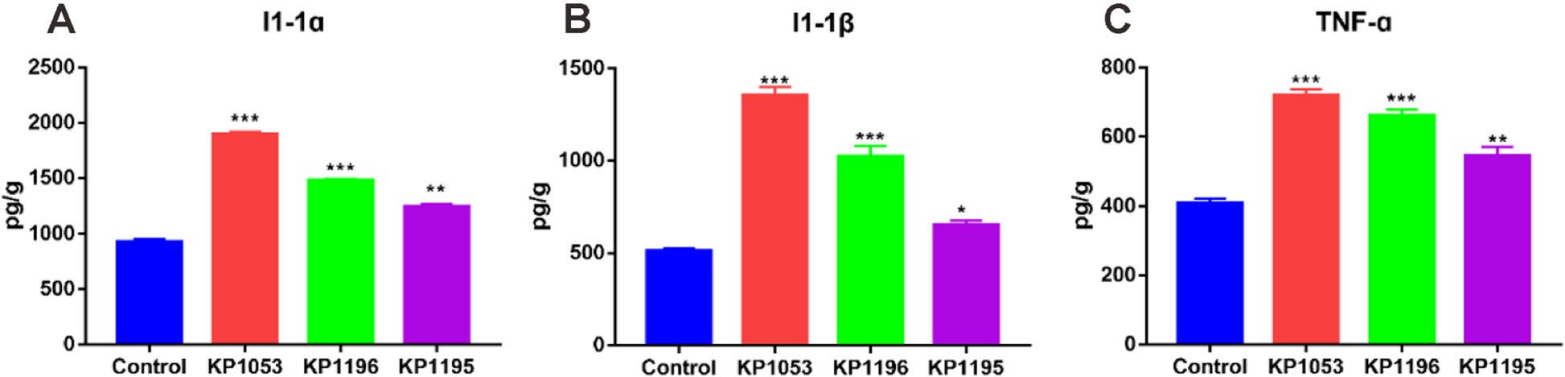
Changes in the content of inflammatory factors in the intestine of zebrafish after infection with Klebsiella pneumoniae of different virulence; 48 h after K. pneumoniae infection, the contents of (**A**) Il-1ɑ, (**B**) Il-1β, and (**C**) TNF-ɑ were determined by ELISA. Error bars are presented as the SEM. Values are presented as the mean ± SEM of three replicates (*P < 0.05, **P < 0.01, ***P < 0.001, and ****P < 0.0001)

ELISA was performed to detect changes in Il-1ɑ, Il-1β, and TNF-ɑ content after infection with the three *K. pneumoniae* strains (Fig. 4). The results showed that the contents of the three cytokines increased significantly after the infection of these three strains of Klebsiella pneumoniae, and the increase was more obvious after KP1053 infection, which increased by up to 2 times. The infection groups of KP1196 and KP1195 increased the cytokine levels between 1.4–1.8 times. These data indicate that the three clinically isolated strains of *K. pneumoniae* can activate intestinal inflammation.

### 16S rRNA gene sequencing to detect the composition of intestinal microbes

**Fig. 5:**
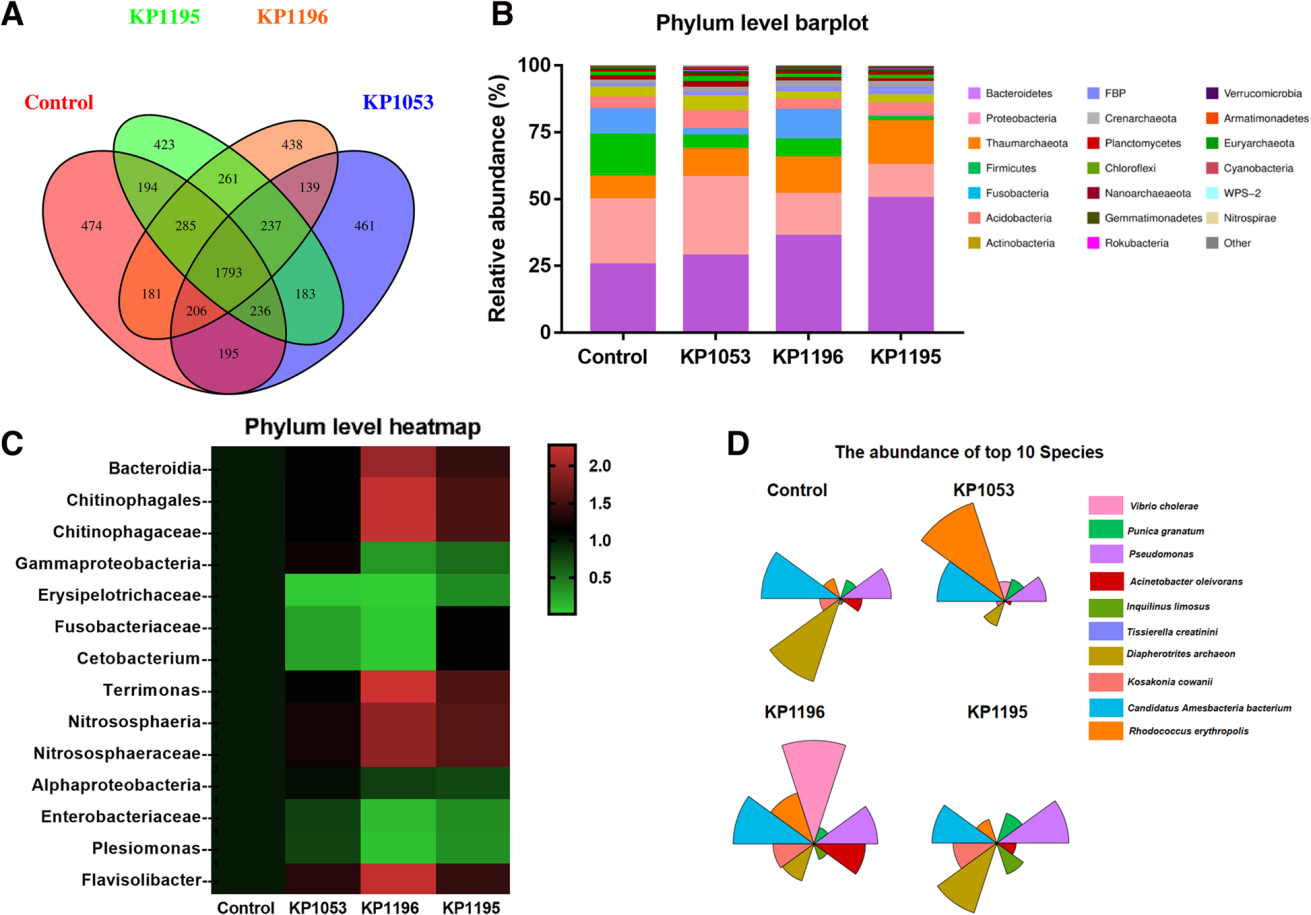
After infecting zebrafish with *Klebsiella pneumoniae* of different virulence, 16S rRNA gene sequencing was used to detect the composition of microorganisms in the intestine. **A** The Venn chart shows the number of OTUs shared and unique to different experimental groups. **B** Profiles of zebrafish gut microbes at the phylum level in different experimental groups. **C** Profiles of zebrafish gut microbes at the top phylum level in different experimental groups. **D** The composition of Top 10 species of zebrafish gut microbes in different experimental groups

To detect the changes in the gut microbial community after infection with the three clinically isolated strains of *K. pneumoniae*, 16S rRNA gene high-throughput sequencing was performed. The results showed that 3564 OTUs were detected in the control group, 3450 OTUs were detected in the KP1053 group, 3540 OTUs were detected in the KP1196 group, and 3612 OTUs were detected in the KP1195 group. The Venn diagrams of the four groups are shown in Fig. 5A. Compared with the control group, the composition of intestinal microbes at the phylum level was significantly different (Fig. 5B). Bacteroidetes were the most abundant among all groups; however, in the KP1195 infection group, the proportion of Bacteroidetes was significantly higher. Proteobacteria was the second most abundant phylum, and their proportion in the KP1053 infection group was significantly higher. Compared with the control group, the proportion of Thaumarchaeota in the three infection groups significantly increased, whereas the proportion of Firmicutes in the KP1053 and KP1195 infection groups significantly reduced.

Analysis at the phylum level showed that Bacteridia increased significantly in the KP1196 infection group, but Erysipelotrichaceae decreased significantly in the three infection groups (Fig. 5C). Among the TOP10 species, in the control group, the species *Diapherotrites archaeon* was the most abundant, but in the KP1053 and KP1196 groups, its abundance was significantly reduced (Fig. 5D). In the KP1053 infection group, the most abundant species was *Rhodococcus erythropolis*. In the KP1096 group, the most abundant species was *Vibrio cholerae*, and the most abundant species in the KP1195-infested group were *Pseudomonas* and *Diapherotrites archaeon*.

### Changes in the Shannon diversity index after infection with the three clinically isolated *K. pneumoniae* strains

**Fig. 6:**
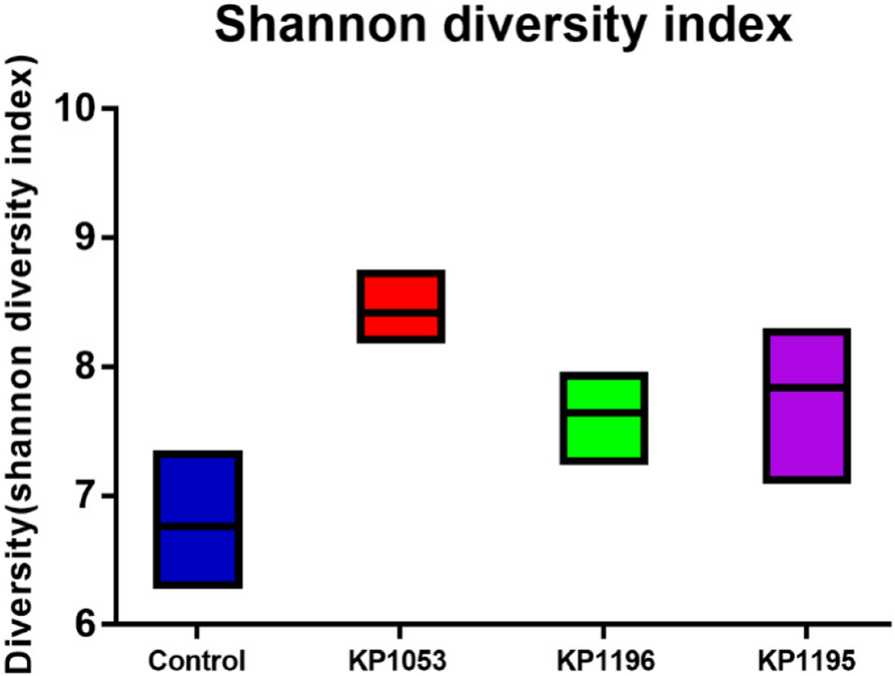
The Shannon index of gut microbial diversity after different strains of *Klebsiella pneumoniae* infected zebrafish

After infection with the three *K. pneumonia* strains, the diversity analysis of intestinal microbes showed that the Shannon diversity index of the three infection groups showed significant increasing changes, indicating significant increasing changes in the microbial diversity of the three infection groups (Fig. 6).

## Discussion

In this study, three clinically isolated strains of *K. pneumoniae* were used to infect adult zebrafish, and the effects on the intestinal tract were investigated. The results showed that the three clinically isolated strains can obviously cause changes in zebrafish intestinal structure and increased inflammation, especially the high virulence resistant strain (KP1053) which caused higher zebrafish intestinal damage. Using 16S rRNA gene sequencing, we showed that all three *K. pneumoniae* strains can cause changes in zebrafish gut microbial diversity, and different strains have different effects.

Zebrafish is a classic model organism to study the pathology and virulence of pathogenic microorganisms [11]. In this study, the same concentration of *K. pneumoniae* was injected into adult zebrafish. The survival rates of zebrafish showed that the virulence-p of these three clinically isolated *K. pneumoniae* strains is KP1053 > KP1096 > KP1095 which is consistent with the results of our previous study using larvae fish. In this study, most zebrafish in the KP1053 group died 72 h after infection. Therefore, subsequent experiments were conducted at 48 h after infection. The results also showed that 48 h of *K. pneumoniae* infection can also cause obvious intestinal damage and physiological changes.

Intestinal goblet cells can play a protective role on intestinal epithelial cells [21]. The infection of many microorganisms can increase the number of goblet cells [22]. The gut is protected by mucus covering the epithelium, which in the small intestine limits the number of bacteria reaching the epithelium, and in the large intestine, the inner mucus layer separates the symbiotic bacteria from the host epithelium. This mucus is produced by goblet cells. In this study, it was found that KP1053 infection can significantly reduce the number of goblet cells However, no such serious damage was observed in the other two experimental groups (KP1196 and KP1195). The decrease of goblet cells may damage the epithelial mucosa and increase bacterial colonization. Studies have shown that the increase of *K. pneumoniae* abundance is proportional to bacterial invasion of epithelial cells.So KP1053 exposed zebrafish have more severe intestinal damage.The mechanism of *K. pneumoniae* colonization of the intestine remains to be completely elucidated.

Inflammation is a body defense mechanism [23]. IL-1ɑ and Il-1β are secreted by macrophages and produce inflammatory responses to external stimuli [24]. TNF-ɑ exerts various functions during the initial immune process [25]. *Klebsiella* has been shown to disrupt the immune system and induce inflammation [26, 27]. In this study, infections of the three strains of *K. pneumoniae* all produced obvious immune defense mechanisms, leading to an increase in cytokine levels. Previous studies have also shown that many microorganisms and toxic compounds can produce significant inflammation after stimulation, and this inflammation can help the body remove xenobiotics [28]. However, excessive inflammation can also cause serious damage to the body [29]. In addition, cytokines have become therapeutic targets for many diseases. For example, in cancer treatment, inflammation and tumor promotion can be inhibited by inhibiting cytokines TNF, Il-1β and Il-16.Therefore, it is important to understand the role of cytokines in Klebsiella pneumoniae infection and their potential use as biomarkers and therapeutic targets in future research.

After infection with the three strains of *K. pneumoniae*, the microbes in the intestines showed obvious changes at the phylum, genus, and species levels. Zebrafish is a vertebrate animal [30], intestinal microbes play a very important role in the normal function of the intestine and the defense of resisting external pathological invasion [31]. The occurrence of many diseases is related to homeostasis changes caused by pathogenic microorganisms in the intestine [32]. In this study, Bacteroidetes and Proteobacteria were found to be the two largest populations of intestinal microbes. A previous study showed that Proteobacteria are the core intestinal microbial population in fish [33], but in the two KP1196 and KP1195 groups, the proportion of proteobacteria was significantly smaller, indicating that the reduction of the normal microbial population will cause more pathology. Bacteroidetes normally reside in the intestines, oral cavity, upper respiratory tract, and reproductive tract of humans and animals [34]. A previous study showed that Bacteroidetes might be related to obesity [35]. The abundance of Bacteroidetes in the three treatment groups was significantly increased. Firmicutes are common in the intestinal microbial population [36]; however, they are significantly reduced in the KP1053 and KP1195 infection groups. This reduction may be caused by the increase in other pathogenic microorganisms. Cyanobacteria are often referred to as blue-green algae [37]. They are highly abundant in zebrafish and can produce toxins and lipopolysaccharides [38]. Both of these substances are toxic and can cause cell necrosis or even tissue disease in zebrafish [38]. In this study, all three groups led to an increase in the Cyanobacteria abundance. These indicate that *K. pneumoniae* enters the intestines of zebrafish, resulting in an increase in the abundance of pathological bacteria. At the genus level, *Vibrio*, *Methylobacterium*, and *Halomonas* were all observed. In addition, at the species level, *Vibrio* was the most abundant intestinal population after KP1196 infection. These findings indicate that *K. pneumoniae* infection increases the abundance of pathological intestinal bacteria.

At the species level, it was found that the most abundant population in the intestine of the non-infected group was *Diapherotrites archaeon*, while in the KP1035 infection group, the most abundant in the intestine was *Rhodococcus erythropolis*. After KP1196 infection, the most abundant species in the intestine was *Vibrio cholerae*. In the KP1195 group, the most abundant intestinal microbe was *Pseudomonas*. This also shows that the different impact of *K. pneumoniae* infections on the microbial diversity in the intestine is different, and this difference is also reflected at the phylum and genus levels. In addition, *K. pneumoniae* increased the Shannon diversity index in the gut of zebrafish. The Venn diagram shows that the gut microbiota shared by normal zebrafish is reduced, producing many microbiota that are not present in the normal gut. Normally, high biodiversity and ecological complexity can maintain the stability of the environment. As a result, Klebsiella pneumoniae processing the extra microbiota may be harmful to the gut and disrupt immune system function. Identifying the responses of individual species is critical to developing precise treatments or prevention methods for microbiome related dysbiosis.

There are many factors affecting the virulence of *K. pneumoniae*, including capsular polysaccharide (CPS) and fimbriae. KP1195 was missing both the CPS synthesis gene (_p_rmpA、_p_rmpA2) and the fimbrial adhesion gene (fimH), and proved to be less virulent. However, colonization of the gut by Klebsiella pneumoniae is contradicted by an established relationship between these factors, such as CPS. KP1196 and KP1053 were similar in virulence, and H&E and the levels of inflammatory factors showed that KP1053 had more severe intestinal damage than KP1195. The results showed that acute intestinal colonization, infection and virulence of these three strains were not completely correlated. Therefore, it is particularly important to further study the pathogenesis of *K. pneumoniae* and find targeted drugs. In addition, patients with colonized *K. pneumoniae* should be strictly and effectively admitted for examination to control the source of infection.

Our study has limitations. While we confirmed the adverse effects of* K. pneumoniae* on the gut, that the specific influencing factors need to be further studied. We were unable to control the number of bacteria in the intestines of zebrafish and did not collect more samples, including due to cost and operational implications. Zebrafish has many advantages as a model organism for studying intestinal colonization, such as a simple and fast process. In a more rigorous and responsible attitude, clinical trial data refer more to mice, rabbits, monkeys and other mammals that are more closely related to humans. Zebrafish experiments are more often used to quickly provide research direction and reveal possible mechanisms.

## Conclusion

*Klebsiella pneumoniae* infection can lead to changes in intestinal pathology and inflammation, and it can also disrupt the diversity of microorganisms in the intestine, causing pathological and physiological changes, which is a clinical diagnosis for *K. pneumoniae* infection in the intestinal tract. Because zebrafish is a model organism that is suitable for high-throughput drug selection, it can provide the possibility for high-throughput screening of therapeutic drugs against clinically isolated *K. pneumoniae*.

### Supplementary Information

**Additional file 1: Supplementary Table 1.** The virulence genes of the isolated K. pneumonia.

**Additional file 2:** **Supplementary Figure 1.** The number of goblet cells per intestinal fold in different treatment groups. (A) Schematic diagram of individual intestinal fold under different treatments. (B) The quantification of goblet cells numbers in per intestinal fold. Error bars are presented as the SEM. Values are presented as the mean ± SEM of three replicates (*P < 0.05, ns: not significant).

## Data Availability

The datasets used and/or analysed during the current study are available from the corresponding author on reasonable request.
