# Supplementary material for: Adult zebrafish infected by clinically isolated Klebsiella pneumoniae with different virulence showed increased intestinal inflammation and disturbed intestinal microbial biodiversity
Source: BMC Infect Dis. 2023 Dec 21;23:899. doi: 10.1186/s12879-023-08766-z (PMC10740243; doi:10.1186/s12879-023-08766-z)
Supplement: Supplementary file 1 — Additional file 1: Supplementary Table 1. The virulence genes of the isolated K. pneumonia. [file 12879_2023_8766_MOESM1_ESM.doc]

Supplementary Table 1. The virulence genes of the isolated K. pneumoniae

| **Characteristic** | KP1053 | KP1196 | KP1195 |
| --- | --- | --- | --- |
| String test | **+** | **+** | **-** |
| **Virulence genes** |  |  |  |
| peg-344 | **+** | **+** | **+** |
| _p_rmpA | **+** | **+** | **-** |
| _p_rmpA2 | **+** | **+** | **-** |
| _c_rmpA | **-** | **-** | **+** |
| iroB | **+** | **+** | **-** |
| iucA | **+** | **+** | **-** |
| ureA | **+** | **+** | **+** |
| uge | **+** | **+** | **+** |
| wabG | **+** | **+** | **+** |
| allS | **+** | **+** | **+** |
| mrkD | **+** | **+** | **+** |
| fimH | **+** | **+** | **-** |
|  |  |  |  |
