# Supplementary material for: Adult zebrafish infected by clinically isolated Klebsiella pneumoniae with different virulence showed increased intestinal inflammation and disturbed intestinal microbial biodiversity
Source: BMC Infect Dis. 2023 Dec 21;23:899. doi: 10.1186/s12879-023-08766-z (PMC10740243; doi:10.1186/s12879-023-08766-z)
Supplement: Supplementary file 2 — Additional file 2: Supplementary Figure 1. The number of goblet cells per intestinal fold in different treatment groups. (A) Schematic diagram of individual intestinal fold under different treatments. (B) The quantification of goblet cells numbers in per intestinal fold. Error bars are presented as the SEM. Values are presented as the mean ± SEM of three replicates (*P < 0.05, ns: not significant). [file 12879_2023_8766_MOESM2_ESM.docx]

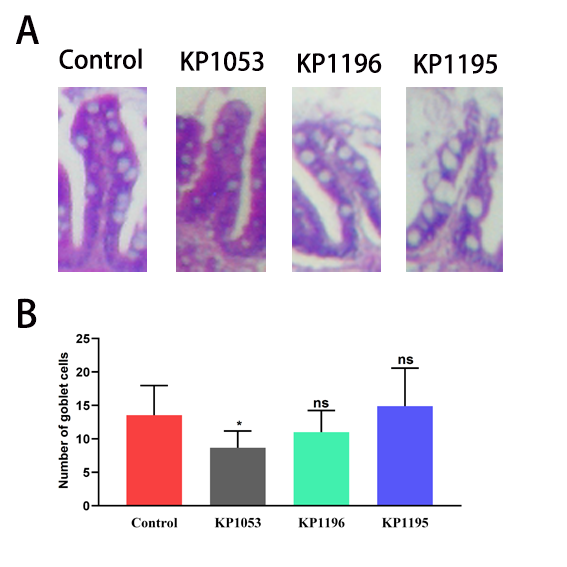


**Supplementary Figure 1** The number of goblet cells per intestinal fold in different treatment groups. (A) Schematic diagram of individual intestinal fold under different treatments. (B) The quantification of goblet cells numbers in per intestinal fold. Error bars are presented as the SEM. Values are presented as the mean ± SEM of three replicates (*P < 0.05, ns: not significant).
